# ACER2 forms a cold tumor microenvironment and predicts the molecular subtype in bladder cancer: Results from real-world cohorts

**DOI:** 10.3389/fgene.2023.1148437

**Published:** 2023-03-01

**Authors:** Jinhui Liu, Chunliang Cheng, Tiezheng Qi, Jiatong Xiao, Weimin Zhou, Dingshan Deng, Yuanqing Dai

**Affiliations:** ^1^ Department of Urology, Xiangya Hospital, Central South University, Changsha City, China; ^2^ National Clinical Research Center for Geriatric Disorders, Xiangya Hospital, Central South University, Changsha, China; ^3^ Xiangya School of Medicine, Central South University, Changsha, China

**Keywords:** ACER2, tumor micro environment, bladder cancer, Chemothearpy, Immunothrapy

## Abstract

**Background:** ACER2 is a critical gene regulating cancer cell growth and migration, whereas the immunological role of ACER2 in the tumor microenvironment (TME) is scarcely reported. Thus, we lucubrate the potential performance of ACER2 in bladder cancer (BLCA).

**Methods:** We initially compared ACER2 expressions in BLCA with normal urothelium tissues based on data gathered from the Cancer Genome Atlas (TCGA) and our Xiangya cohort. Subsequently, we systematically explored correlations between ACER2 with immunomodulators, anti-cancer immune cycles, tumor-infiltrating immune cells, immune checkpoints and the T-cell inflamed score (TIS) to further confirm its immunological role in BLCA TME. In addition, we performed ROC analysis to illustrate the accuracy of ACER2 in predicting BLCA molecular subtypes and explored the response to several cancer-related treatments. Finally, we validated results in an immunotherapy cohort and Xiangya cohort to ensure the stability of our study.

**Results:** Compared with normal urinary epithelium, ACER2 was significantly overexpressed in several cell lines and the tumor tissue of BLCA. ACER2 can contribute to the formation of non-inflamed BLCA TME supported by its negative correlations with immunomodulators, anti-cancer immune cycles, tumor-infiltrating immune cells, immune checkpoints and the TIS. Moreover, BLCA patients with high ACER2 expression were inclined to the luminal subtype, which were characterized by insensitivity to neoadjuvant chemotherapy, chemotherapy and radiotherapy but not to immunotherapy. Results in the IMvigor210 and Xiangya cohort were consistent.

**Conclusion:** ACER2 could accurately predict the TME and clinical outcomes for BLCA. It would be served as a promising target for precision treatment in the future.

## 1 Introduction

Bladder cancer (BLCA) is the second common urinary malignancy with 81,180 new cases each year, and results in 17,100 deaths in United States (Siegel et al., 2022). Despite multiple treatment strategies including surgery, chemotherapy and radiotherapy, have been applied in the present, half of BLCA patients would still relapse or found to be metastasized after the radical cystectomy (Witjes et al., 2021). And the prognosis of metastatic BLCA is still frustrating.

Recently, cancer immunotherapy represented by immune checkpoint blockade (ICB) had gained colossal survival benefits for advanced BLCA (Bellmunt et al., 2017; Powles et al., 2018). However, BLCA varied with significant heterogeneity, thus a portion of patients were observed not to respond to ICB (Rosenberg et al., 2016; Sharma et al., 2017). A main mechanism is that lower neoantigen burden and tumor mutation burden in TME suppresses the response to ICB due to the impair of T-cells to destroy tumor cells (Morad et al., 2021). Besides, tumor microenvironment (TME) was vital for the effect of immunotherapy, in which tumor cells themselves can upregulate the expression of PD-L1 and stimulate the expression of PD-L1 in TME cells, and thus suppresses antitumor immune response of cytotoxic T-cells (Alsaab et al., 2017). ICB inhibited tumor growth by re-invigorating tumor-cytotoxic T-cells. Unfortunately, non-inflamed TME would cause resistance to ICB by some molecules or pathways. Consequently, it was critical to explore new TME state indicators and treatment response biomarkers for BLCA to early screen a suitable group who may respond to ICB.

Alkaline ceramidase 2 (ACER2) was a sphingolipid metabolizing enzyme localized to the Golgi complex, which could convert ceramide to sphingosine *in vivo* (Sun et al., 2010). ACER2 was highly expressed in a majority of human tumor tissues (Xu et al., 2006; Xu et al., 2018a). Accumulated studies have reported that ACER2 was transactivated to mediate the DNA damage response, and regulated autophagy and programmed cell death by increasing the production of reactive oxygen species (ROS) (Xu et al., 2016; Wang et al., 2017; Xu et al., 2018a). Besides, ACER2 was observed to be a crucial biomarker, which contribute to the tumor growth, invasion, and migration in several cancers (Liu et al., 2020; Zhang et al., 2020; Zheng et al., 2022). Considering the effect in the cancer cell apoptosis and proliferation, ACER2 could be served as a potential molecular target for cancer treatment.

However, the immunological role of ACER2 in tumor microenvironment (TME) was rarely reported. Herein, we comprehensively explored the relationship between ACER2 expression and TME in the BLCA. We found that ACER2 promoted the development of a non-inflamed BLCA TME, and had the potential to predict the molecular subtypes of BLCA.

## 2 Methods

### 2.1 Data collection and preprocessing

We obtained the mRNA expression data (FPKM) value and corresponding clinicopathologic information of bladder cancer in The Cancer Genome Atlas (TCGA) (https://portal.gdc.cancer.gov/). The cohort comprised 410 BLCA samples and 19 normal urothelium tissues. And then, the FPKM value of TCGA cohort was translated into transcripts per kilobase million (TPM) value before analysis.

The validation cohort was derived from the bladder cancer patients who underwent surgery in Xiangya Hospital. It comprised a total of 57 BLCA cancer samples and 13 normal urothelium tissues. Fresh tissues were collected and stored with liquid nitrogen immediately. First, total RNA was extracted from fresh tissues using TRIzol (Invitrogen, Carlsbad, California, United States). The total RNA was then quantified using NanoDrop and Agilent 2100 biological analyzers (Thermo Fisher Scientific, MA, United States). After constructing the mRNA library, we further purified and fragmented the total RNA into small pieces. After that, we synthesized the first-strand cDNA and the second-strand cDNA, and further amplified by PCR to construct the final library (single-stranded circular DNA). Finally, 57 BLCA samples and 13 normal tissues were qualified and sequenced on the BGISEQ-500 platform (BGI-Shenzhen, China).TPM value was also translated in Xiangya cohort. Besides, the data has been uploaded to the GEO database (GSE188715).

IMvigor210 was an immunotherapy cohort in which BLCA patients received anti-PD-1 therapy. We obtained the mRNA expression data and corresponding clinicopathologic information from http://research-pub.Gene.com/imvigor210corebiologies/based on the Creative Commons 3.0 License.

### 2.2 Depicting immunological characteristics of TME

The effect of anticancer immunity was highly associated with the expression of immunomodulators, activity of the cancer immunity cycle, infiltration level of tumor infiltrating lymphocytes (TILs), and the expression of inhibitory immune checkpoints in an inflamed tumor microenvironment (TME). We collected the information of 122 immunomodulators in the previous study (Charoentong et al., 2017), and compared differential expression immunomodulators including MHC, receptors, chemokines, and immune stimulators between low and high ACER2 groups. We further explored the effect of ACER2 in impacting cancer immunity cycle in BLCA. The cancer immunity cycle was stepwise events proceed and expand iteratively comprising in 7 critical steps, which determined the fate of tumor cells (Chen and Mellman, 2013). Thereafter, we calculated the correlation between the infiltration level of TILs in TME and ACER2 expression using seven independent algorithms, including Cibersort-ABS, MCP-counter, quanTIseq, TIMER, xCell, TIP, and TISIDB (Newman et al., 2015; Becht et al., 2016; Li et al., 2016; Xu et al., 2018b; Finotello et al., 2019; Ru et al., 2019; Li et al., 2020). The relationships between ACER2 and the corresponding effector genes of these TILs were also analyzed. Moreover, we correlated the ACER2 expression with 22 common immune checkpoint inhibitors (ICIs), such as PD-1, PD-L1, CTLA-4, and LAG-3. Finally, we analyzed the T-cell inflamed score (TIS) and corresponding TIS-related effector genes in the TME, which represented pre-existing cancer immunity and predicted the clinical response of ICB (Ayers et al., 2017).

### 2.3 Prediction to molecular subtypes and therapeutic response

Consider that BLCA varied with high heterogeneity and differed distinctly in the treatment response and prognosis, several molecular subtype systems of BLCA had been constructed (Sjödahl et al., 2022), including UNC, Baylor, TCGA, MDA, Lund, CIT, and Consensus subtype systems. We used ConsensusMIBC and BLCA subtyping R packages to determine the molecular subtype systems, and depicted the relationship between the specific signatures of molecular subtypes and ACER2 expression (Hu et al., 2021). Receiver operating characteristic (ROC) curves were used for evaluating the accuracy of ACER2 in predicting BLCA molecular subtypes. The difference of neoadjuvant chemotherapy related mutation was evaluated in the high and low ACER2 groups. Subsequently, we explored several therapeutic responses to immunotherapy, targeted therapies and radiotherapy. Finally, drug-target genes were collected and analyzed in the Drug Bank database.

### 2.4 Real-time quantitative PCR (qPCR)

Bladder cancer and normal bladder cell lines were used to extracted total RNA using cell total RNA isolation kit (Foregene, China) according to manufacturer’s protocol. cDNA was synthesized using UeIris II RT-PCR System for First-Strand cDNA Synthesis (US Everbright, China). qRT-PCR was performed using SYBR Green qPCR Master Mix (US Everbright, China) on CFX Connect System (Bio-Rad, United States). Gene expression levels were normalized to the “housekeeping” gene GAPDH. The primers were designed and synthesized by Sangon Biotech (Shanghai, China) and detailed primer sequences were listed below: ACER2: (forward primer: 5′- CCT​TTG​GGT​TCT​GAT​GTG​TGC​TTT​G-3′; reverse primer: 5′-GGA​CAC​TGA​CCA​CCA​CCT​TGA​AC-3′); GAPDH: (forward primer: 5′- CAA​GGC​TGT​GGG​CAA​GGT​CAT​C-3′; reverse primer: 5′- GTG​TCG​CTG​TTG​AAG​TCA​GAG​GAG-3′).

### 2.5 Statistical analysis

Pearson or Spearman coefficients was calculated to explore correlations between variables. For variables fitting a normal distribution between binary groups, t-test was used to compare the differences. For categorical variables, chi-squared test or Fisher’s exact test was performed. Analyses with two-sided *p* = 0.05 were considered as the threshold of statistical significance. Receiver operating characteristic (ROC) curves were depicted to evaluate the predictive accuracy for molecular subtypes. All the statistical analyses and visualizations were performed in R software, Version: 4.2.2.

## 3 Result

### 3.1 Pan-cancer analysis evaluates the immunological role of ACER2

**FIGURE 1:**
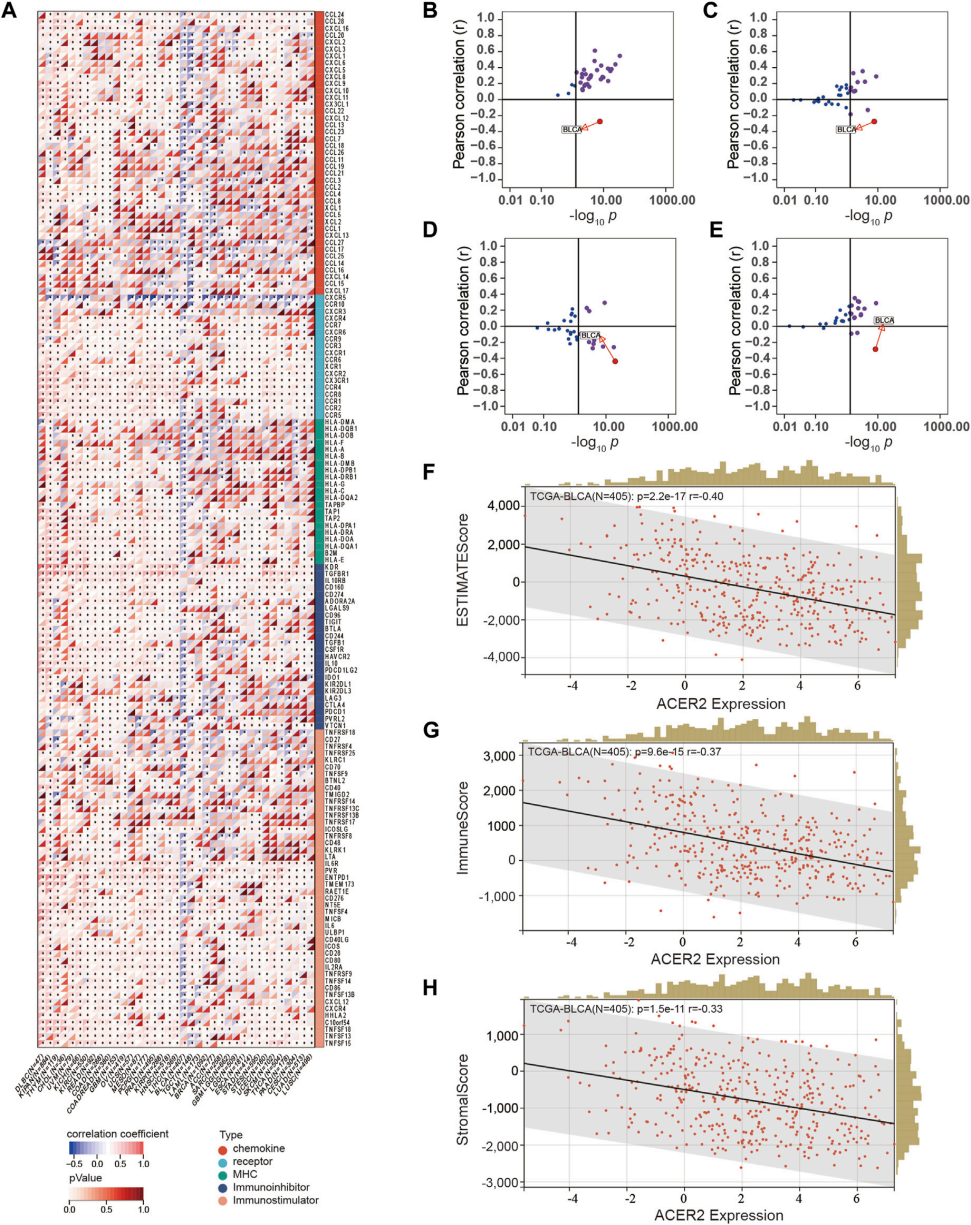
Correlations between ACER2 with immunological characteristics in pan-cancers. **(A)** Correlation between ACER2 and 122 immunomodulators (chemokines, receptors, MHC, and immunostimulators). **(B–E)** Correlation between ACER2 and four immune checkpoints, namely, PD-L1, CTLA-4, PD-1, and LAG-3. **(F–H)** Correlation between ACER2 expression and the ESTIMATE score, Immune score and Stromal score in TME.

We performed pan-cancer analysis to illustrate the immunological role of ACER2, and screened cancer types which were impacted most by ACER2. Figure 1A revealed the relationship between ACER2 expression and immunomodulators across 37 cancer types. We found that ACER2 was positively associated with multiple immunomodulators in several cancer types, such as THYM, CHOL, ACC, and SARC. Remarkably, we noticed negative correlations in BLCA between ACER2and a majority of immunomodulators including immunomostimulators, MHC, receptors, and chemokines. Likewise, a negative relation was also observed in breast cancer. We then explored the relationship between ACER2 expression and several critical immune checkpoints. Of note, ACER2 expression in BLCA was negatively correlated with four immune checkpoints, namely, PD-L1, PD-1, CTLA-4, and LAG-3 (Figures 1B–E). Besides, we uncovered ACER2 expression in BLCA was negatively associated with the ESTIMATE score, Immune score and Stromal score in TME (Figures 1F–H). In brief, ACER2 was considered potential to be a biomarker to predict the TME status especially in BLCA. High ACER2 expression in BLCA may contribute to the formation of non-inflamed TME, as the result of the reduced level of immunomodulators, immune checkpoints, immune cells, and stromal cells in TME.

### 3.2 ACER2 is highly expressed in BLCA

**FIGURE 2:**
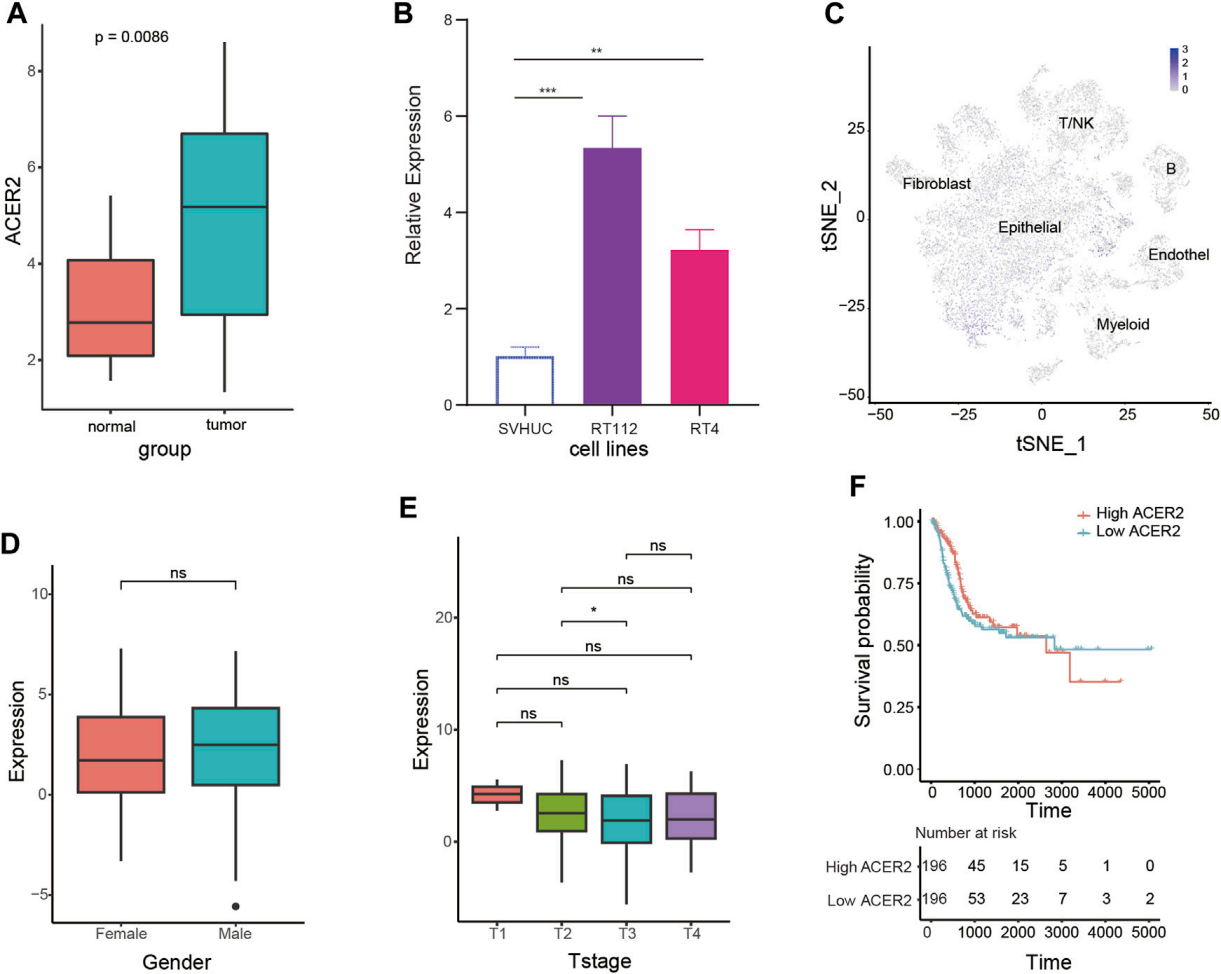
Expression characteristics of ACER2 in BLCA. **(A)** Expression analysis of ACER2 in the Xiangya cohort. **(B)** Expression of ACER2 in diverse cell lines. **(C)** Single-cell analysis of ACER2 expression in tumor and normal tissues. **(D, E)** Correlations between ACER2 expression and gender, T stage. **(F)** Kaplan-Meier curve for disease-specific survival by expression of ACER2.

We assessed the expression characteristics of ACER2 in BLCA. The qPCR analysis of Xiangya cohort revealed that ACER2 was significantly elevated in tumor tissues (*p* = 0.0086) compared with normal urothelium (Figure 2A). Then we analyzed the differential expression of ACER2 in diverse cell lines. ACER2 was significantly higher expressed in bladder cancer cell lines in comparison to bladder epithelial cell lines (Figure 2B). Besides, single-cell analysis also indicated a higher expression in the malignant epithelial cells (Figure 2C). However, we did not observe the correlations between ACER2 expression and gender, T stage (Figures 2D,E). No significant difference was observed in the disease-specific survival between high- and low-ACER2 (*p* = 0.088) (Figure 2F).

### 3.3 ACER2 contributes to a non-inflamed TME in BLCA

**FIGURE 3:**
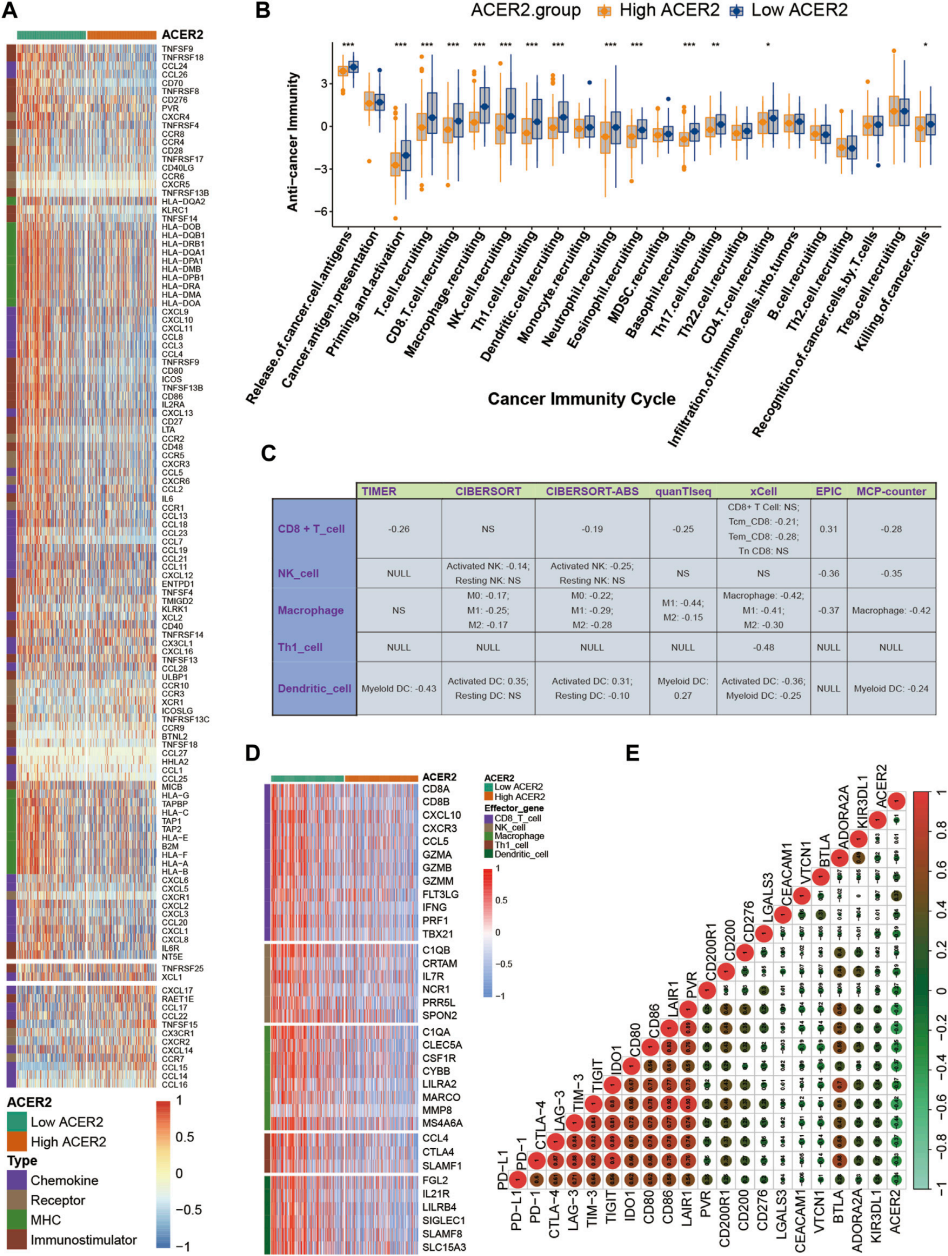
ACER2 correlated with the tumor immune microenvironment in BLCA. **(A)** Differential expression of ACER2 between high- and low-ACER2 tissues in BLCA. **(B)** Differential expression of ACER2 among various steps of the anti-tumor immune cycle. **(C)** Correlation between ACER2 and the infiltration levels of five TIICs with various algorithms. **(D)** Differential expression of effector genes of five mentioned TIICs between high- and low-ACER2 tissues in BLCA. **(E)** Correlation betweenACER2 and 20 inhibitory immune checkpoints.

ACER2 exhibited negative correlations with multiple immunomodulators (Figure 3A). The high ACER2 group had an obvious downregulation of the majority of chemokines including CCL4, CCL3, CCL24, and CCL26. Immunostimulators including CD80, CD86, ICOS, and TNFSF13B were also found negatively correlated with ACER2. Subsequently, four major steps in the anti-cancer immunity cycle were downregulated in the high ACER2 group (Figure 3B), which included the release of cancer cell antigens, priming and activation, immune cells recruiting and killing of cancer cells. Strikingly, we noticed that recruiting of multiple immune cells such as T-cell, CD8^+^ T-cell, macrophage cell, NK cell, Th1 cell, dendritic cell, neutrophil cell, eosinophil cell, basophil cell, Th17 cell, CD4 T-cell were significantly restrained in the high ACER2 group. It implied the reduction of infiltration levels of effector TILs in the TME.

**FIGURE 4:**
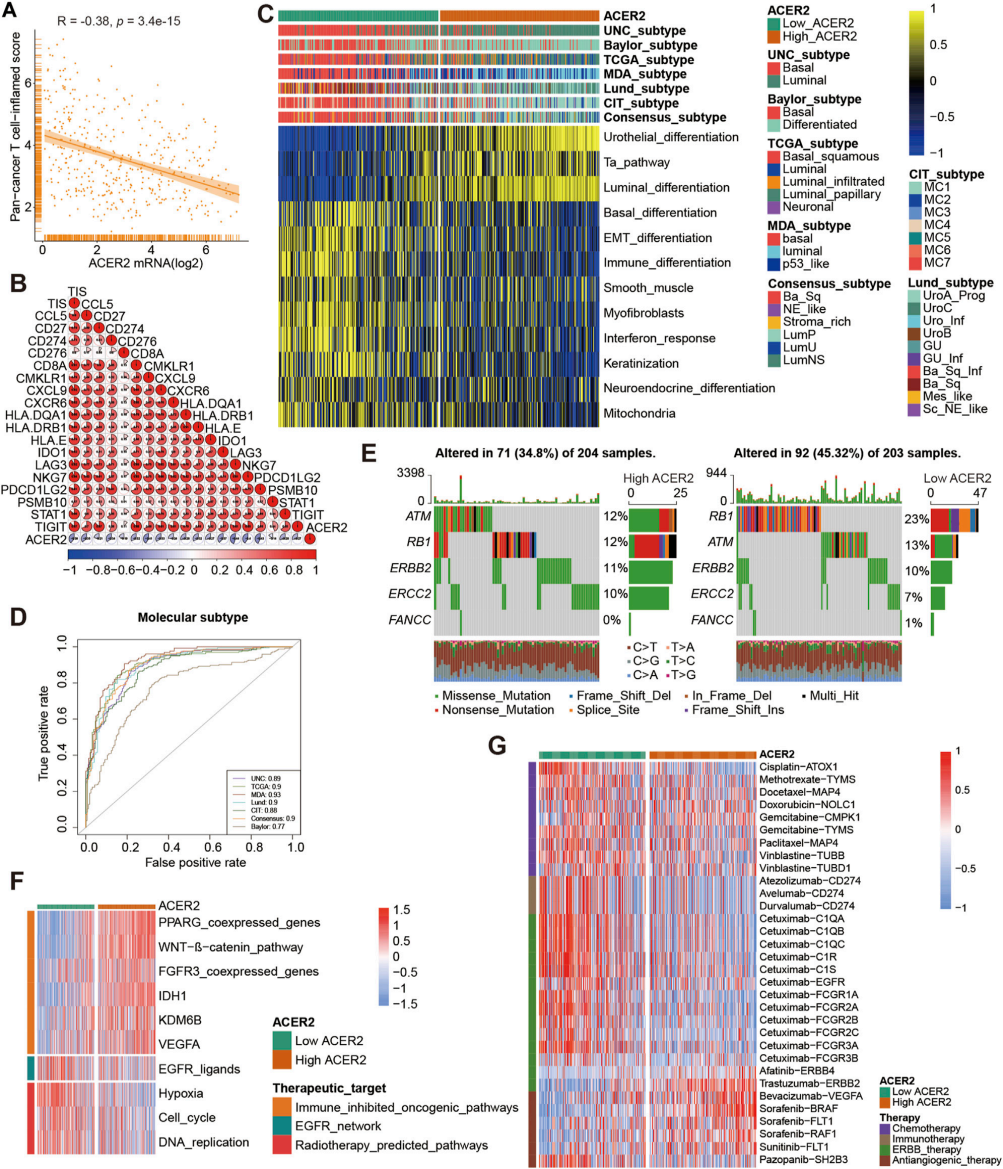
ACER2 predicts the molecular subtype and response to several therapies in BLCA. **(A, B)** Correlation between ACER2 with TIS and the corresponding TIS-related effector genes. **(C)** Correlations between ACER2 and molecular subtypes with seven different subtyping systems. **(D)** ROC analysis on the prediction accuracy of ACER2 for molecular subtypes with different systems. **(E)** Mutational profiles of neoadjuvant chemotherapy-related genes in low- and high-ACER2 tissues. **(F)** Correlation between ACER2 and the enrichment scores of therapeutic signatures. **(G)** Correlation between ACER2 and the drug-target genes of various therapies.

For further validating the correlation between ACER2 and TILs in the TME, we calculated the infiltration level of TILs using seven independent algorithms. Similarly, ACER2 was negatively correlated with the infiltration level of CD8^+^ T-cell, macrophage, NK cell, Th1 cell, and dendritic cell (Figure 3C). Likewise, ACER2 was proved to be negatively correlated with the effector genes of TILs (Figure 3D). Moreover, we explored the relation between ACER2 and immune checkpoint inhibitors in the TME. ACER2 was found to be negatively correlated with a majority of usual immune checkpoint inhibitors including PD-L1, PD-1, CTLA-4, LAG-3, TIM-3, and TIGIT (Figure 3E). Finally, we further discovered ACER2 was negatively correlated with the TIS and corresponding TIS-related effector genes (Figures 4A,B). In summary, the results revealed that ACER2 contributed to the formation of non-inflamed TME in BLCA.

### 3.4 ACER2 predicts molecular subtypes and drug sensitivity

BLCA was a highly heterogeneous malignancy with various molecular subtypes which mainly differed in sensitivity to diverse therapeutic regimens. Therefore, we distinguished BLCA molecular subtypes in the TCGA within ACER2 expression. Figure 4C illustrated patients with low ACER2 expression were more likely to be basal subtype and characterized by basal differentiation, EMT differentiation, immune differentiation, and keratinization. As shown in previous studies, basal subtype was more sensitive to ICB with higher immune infiltration level and pathological response rates, which was consistent to our prior finding (Kamoun et al., 2020; Necchi et al., 2020). Thus, patients with lower ACER2 expression may benefit from ICB. Conversely, individuals with high ACER2 expression were inclined to luminal subtype with urothelial differentiation, Ta pathway, and luminal differentiation. Thereafter, ROC analysis was adopted to evaluate the predictive accuracy of ACER2 for molecular subtypes, and the area under the ROC curves was calculated ranging from 0.77 to 0.93 (Figure 4D).

In order to further explore the correlation between ACER2 with neoadjuvant chemotherapy (NAC), we found that low ACER2 group was more likely to carry neoadjuvant chemotherapy related mutation such as RB1 (23%), ATM (13%), and ERBB2 (10%). We also found that high mutation rates of ATM (12%), RB1 (12%), and ERBB2 (11%) in the high ACER2 group (Figure 4E). Notably, chemotherapy related mutation of RB1 was significantly higher in the low ACER2 group, which implied that tumors with low ACER2 may be more sensitive to NAC. Ulteriorly, we revealed enrichment scores for radiotherapy-predicted pathways and EGFR ligands was higher in the low ACER2 group (Figure 4F). And several enrichment scores for various immunosuppressive oncogenic pathways such as PPARG coexpressed genes, WNT-β-catenin pathway, and IDH1 were significantly higher in high ACER2 group, which also indicated the formation of non-inflamed TME in BLCA. Besides, we used the Drugbank database to determine that the low ACER2 group was sensitive to immunotherapy and ERBB therapy, whereas antiangiogenic therapy may be more suitable for high ACER2 group (Figure 4G). Collectively, neoadjuvant chemotherapy, adjuvant chemotherapy, immunotherapy, ERBB therapy could be used for patients with low ACER2.

### 3.5 Validation of the roles of ACER2 in the Xiangya cohort

**FIGURE 5:**
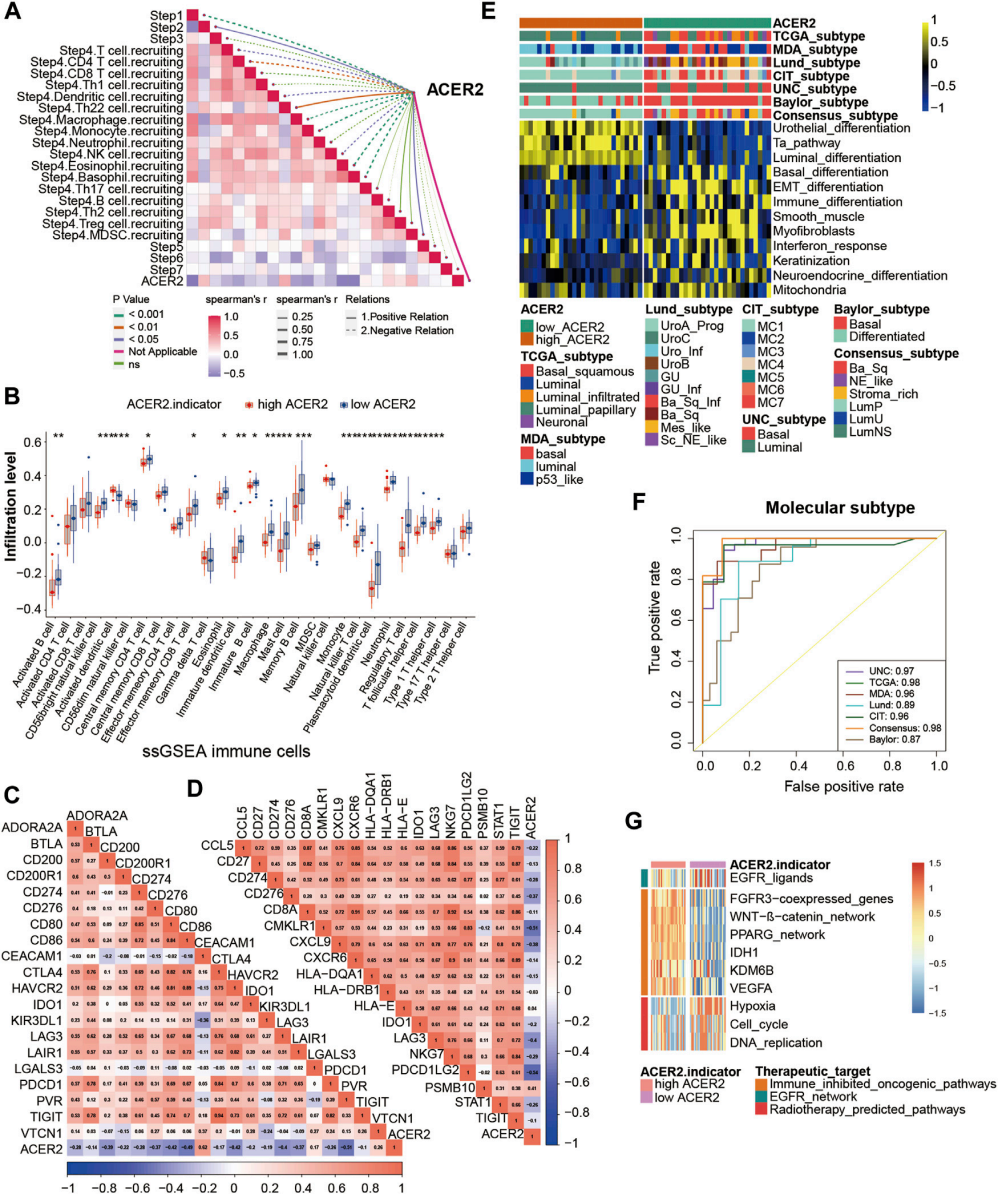
Validation of the ACER2 prediction for molecular subtypes and the response to several therapies in the Xiangya cohort. **(A)** Correlations between ACER2 and the process of the anti-cancer immunity cycle. **(B)** Correlations between ACER2 and multiple ssGSEA immune cells. **(C)** Correlations between ACER2 and 20 immune checkpoints. **(D)** Correlation between ACER2 and TIS-related effector genes. **(E)** Correlations between ACER2 and seven molecular subtype systems. **(F)** ACER2 molecular subtype prediction accuracy. **(G)** Correlation between ACER2 and the enrichment scores of therapeutic signatures.

Thereafter, we further validated the role of ACER2 in Xiangya cohort. ACER2 negatively correlated with the several critical steps of the anti-cancer immune cycles especially including the release of cancer cell antigens, and immune cells recruiting (Figure 5A). Consistently, ACER2 was also negatively associated with multiple ssGSEA immune cells including activated dendritic cell, macrophage cell, natural killer cell, regulatory T-cell, T follicular helper cell (Figure 5B). Subsequently, a negative relationship between ACER2 and immune checkpoints was observed in Xiangya cohort (Figure 5C). Besides, we confirmed that lower ACER2 was associated with high expression in T-cell inflamed scores effector genes including CD274, LAG-3, and TIGHT (Figure 5D). Consistent with the prediction for subtype in TCGA, high ACER2 group was more likely to be luminal subtype in Xiangya cohort (Figure 5E), and the accuracy of ACER2 in prediction for molecular subtypes was over 0.87 (Figure 5F). Likewise, high ACER2 was more likely to respond to immune inhibited oncogenic therapy (Figure 5G).

### 3.6 ACER2 predicted clinical response of ICB

**FIGURE 6:**
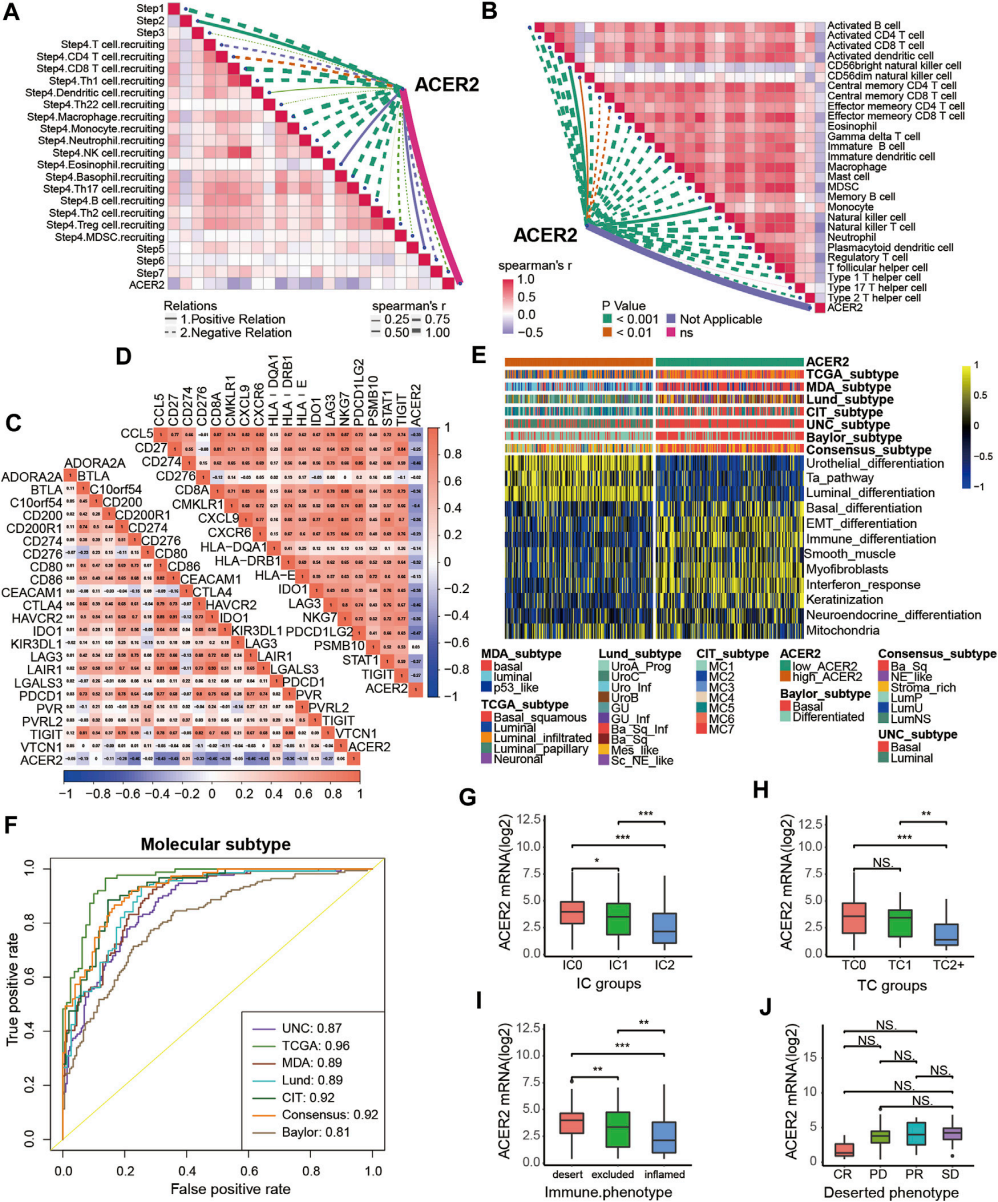
Validation of the ACER2 prediction for low immune infiltration and molecular subtypes in the IMvigor210. **(A)** Correlation between ACER2 and the process of the anti-cancer immunity cycle. **(B)** Correlation between ACER2 and several immune-related cells. **(C)** Correlation between ACER2 and ICI effector genes. **(D)** Correlation between ACER2 and TIS-related genes. **(E, F)** Correlations between ACER2 and seven molecular subtype systems and corresponding ROC analysis for prediction accuracy. **(G)** Differential expression of ACER2 in different IC groups. **(H)** Differential expression of ACER2 in different TC groups. **(I)** Differential expression of ACER2 in three immune phenotypes (the desert, excluded, and inflamed). **(J)** Correlation between ACER2 and the clinical response of tumor immunotherapy in the desert group.

We further explored the effect of ACER2 in predicting clinical response to ICB in an immunotherapy cohort, the IMvigor210 cohort, in which patients received anti-PD-1 therapy. In the IMvigor210 cohort, ACER2 was also observed negative correlations with several critical steps of cancer immune cycles (Figure 6A), thus the infiltration level of TILs in TME was downregulated when ACER2 high expressed (Figure 6B). Whereafter, we found that ACER2 was negatively correlated with the expression of several ICI genes and the TIS genes (Figures 6C,D). In addition, high expressions of ACER2 were inclined to the luminal subtype which was consistent with the results captured from the TCCA cohort (Figure 6E). And the area under ROC curves of predictive accuracy for molecular subtypes was calculated ranging from 0.81 to 0.96 (Figure 6F).

In the IMvigor210 cohort, immunohistochemistry (IHC) was performed to detect the PD-L1 expression on immune cells or cancer cells. Based on the PD-L1 expression level, immune cells and cancer cells were classified into three groups. We discovered ACER2 was significantly expressed in the TC0 and IC0 groups (Figures 6G,H), which existed the lowest PD-L1 expression in the immune cells and cancer cells, respectively. And ACER2 was higher expressed in the desert TME compared with inflamed TME (Figure 6I). Moreover, we compared the ACER2 expressions in different clinical responses to ICB. ACER2 expression was lower in patients with desert TME with complete response (CR) compared with partial response (PR), progressive disease (PD), and stable disease (SD). Even so, we did not observe significant difference across these groups (Figure 6J).

## 4 Discussion

In this study, we uncovered that ACER2 was a potential molecular biomarker to reflect the TME status in diverse cancers especially in BLCA, and contribute to the formation of non-inflamed TME in BLCA. The expression of ACER2 would be a convincing indicator to predict the molecular subtypes. Patients with low ACER2 expression were more likely to be basal subtype, and sensitive to ICB with higher immune infiltration levels. Besides, individuals with lower ACER2 expression were more likely to response to neoadjuvant chemotherapy, adjuvant chemotherapy and ERBB therapy.

Sphingolipid metabolism is mainly induced by ceramidases and plays essential roles in numerous human diseases, including cancer. Ceramidases are a family comprising acid ceramidase, neutral ceramidase, and alkaline ceramidase 1, 2, and 3, which are encoded by ASAH1, ASAH2, ACER1, ACER2 and ACER3, respectively. (Parveen et al., 2019). Ceramidases regulates ceramide catabolism by converting ceramide into sphingosine (SPH). Afterwards, SPH can be further phosphorylated to sphingosine-1 phosphate (S1P). Multiple previous studies revealed ceramide was associated with stress-related cellular responses and apoptosis, and S1P was related to cell proliferation, and tissue regeneration (Perry et al., 1996; Spiegel et al., 1998; Sassoli et al., 2019). The expression of ceramidases balanced the ceramide, SPH, and S1P levels, and substantially contributed to modulation of cell fate (Van Brocklyn and Williams, 2012).

ACER2 was a transcriptional target of p53, and the activation of ACER2 by p53 mediated DNA damage response by increasing the production of ROS (Xu et al., 2016; Xu et al., 2018a). The overexpression of ACER2 in multiple cancer cells regulated proliferation, DNA damage response, programmed cell death, and autophagy. Zhang et al. demonstrated that ACER2 enhanced TIM-mediated promotive effects of cancer cell growth and mitochondrial respiration in ER-positive breast cancer (Zhang et al., 2020). Meanwhile, ACER2 was revealed to promote the growth, invasion, and migration of hepatocellular carcinoma cells (Liu et al., 2020). Whereas, a current investigation revealed a lower ACER2 elevated gastric cancer cell proliferation and migration ability, and thus inhibited apoptosis (Zheng et al., 2022). In our study, we uncovered ACER2 was overexpressed in the tumor tissues and tumor cell lines of BLCA. The relatively higher expression of ACER2 inhibited the killing of tumor cells by decreasing TILs in the TME. Besides, ACER2 was negatively correlated with a majority of common immune checkpoint inhibitors, which restrained the re-invigorating of tumor-cytotoxic T-cells that recognized and eradicated cancer cells. Collectively, ACER2 contributed to the formation of non-inflamed TME in BLCA, hence ACER2 may also contribute to the tumor growth, invasion, metastasis in the BLCA. Unfortunately, we did not observe the difference of ACER2 expression in T stage, prognostic benefit and clinical response to ICB. Although no statistical difference was observed in clinical response to ICB, there was a significant increasing trend. Besides, the insignificance was occurred due to a relatively small sample size, and need to verify in further larger-scale cohorts. Moreover, they would also be partly explained by the dual role of ectopic ACER2 in tumor cell proliferation and death. Xu et al. uncovered that ACER2 promoted the generation of S1P and S1P-mediated cell proliferation and survival, whereas the overexpression of ACER2 may also cause cell growth arrest as the result of an accumulation of sphingosine (Xu et al., 2006). Taken together, we discovered the suppressive immunological role of ACER2 in TME, but precise effects of ACER2 on tumor growth and death were perhaps controlled by multiple factors.

In multiple cancers, S1P produced by ceramidase played a crucial role in tumor growth and resistance to chemotherapy, thus several inhibitors targeting the ceramidase had been developed. Bhabak et al. modified the structural of previous ceramidase inhibitors, and discovered an enhancement of apoptotic cell death in breast cancer cell lines by inhibiting ceramidase (Bhabak et al., 2013). In addition, Carmofur, an ASAH1 inhibitor, was approved to be against colorectal cancer in Japan (Parveen et al., 2019). It indicated inhibition of ceramidases was potential treatment which induced apoptosis, and elevated the response to chemotherapy. Despite up to now there were no inhibitors targeting ACER2, we identified the potential response to drugs based on the ACER2 expression in BLCA. According to the ACER2 expression, BLCA patients could be divided into basal and luminal subtypes. Individuals with lower ACER2 expression were more likely to respond to ICB, chemotherapy, and ERBB therapy which contributed to precision medicine.

The study inevitably existed several limitations. Firstly, all the results were performed based on bioinformatic analyses without *in vivo* or *in vitro* experiments to explore mechanism. Secondly, despite the result was validated well in our Xiangya cohort, the total sample size (only 57) was an inevitable flaw. Thirdly, we used the median ACER2 mRNA expression as the threshold value to distinguish high or low expression groups without determining the optimal cut-off value. Hence, validation with more data from tumor tissues and experiments are imperatively needed.

## 5 Conclusion

In summary, we discovered that ACER2 promoted the formation of non-inflamed TME in BLCA which was resistant to cancer immunotherapy. Meanwhile, ACER2 could be used as a convincing indicator to predict the molecular subtypes and indicate sensitive treatment options.

## Data Availability

The original data presented in this study has been uploaded to the GEO database (GSE188715). Further inquiries can be directed to the corresponding authors.

## References

[B1] Alsaab H. O. Sau S. Alzhrani R. Tatiparti K. Bhise K. Kashaw S. K. (2017). PD-1 and PD-L1 checkpoint signaling inhibition for cancer immunotherapy: Mechanism, combinations, and clinical outcome. Front. Pharmacol. 8, 561. 10.3389/fphar.2017.00561 28878676 PMC5572324

[B2] Ayers M. Lunceford J. Nebozhyn M. Murphy E. Loboda A. Kaufman D. R. (2017). IFN-γ-related mRNA profile predicts clinical response to PD-1 blockade. J. Clin. Invest. 127 (8), 2930–2940. 10.1172/JCI91190 28650338 PMC5531419

[B3] Becht E. Giraldo N. A. Lacroix L. Buttard B. Elarouci N. Petitprez F. (2016). Estimating the population abundance of tissue-infiltrating immune and stromal cell populations using gene expression. Genome Biol. 17 (1), 218. 10.1186/s13059-016-1070-5 27765066 PMC5073889

[B4] Bellmunt J. de Wit R. Vaughn D. J. Fradet Y. Lee J. L. Fong L. (2017). Pembrolizumab as second-line therapy for advanced urothelial carcinoma. N. Engl. J. Med. 376 (11), 1015–1026. 10.1056/NEJMoa1613683 28212060 PMC5635424

[B5] Bhabak K. P. Kleuser B. Huwiler A. Arenz C. (2013). Effective inhibition of acid and neutral ceramidases by novel B-13 and LCL-464 analogues. Bioorg Med. Chem. 21 (4), 874–882. 10.1016/j.bmc.2012.12.014 23312611

[B6] Charoentong P. Finotello F. Angelova M. Mayer C. Efremova M. Rieder D. (2017). Pan-cancer immunogenomic analyses reveal genotype-immunophenotype relationships and predictors of response to checkpoint blockade. Cell Rep. 18 (1), 248–262. 10.1016/j.celrep.2016.12.019 28052254

[B7] Chen D. S. Mellman I. (2013). Oncology meets immunology: The cancer-immunity cycle. Immunity 39 (1), 1–10. 10.1016/j.immuni.2013.07.012 23890059

[B8] Finotello F. Mayer C. Plattner C. Laschober G. Rieder D. Hackl H. (2019). Molecular and pharmacological modulators of the tumor immune contexture revealed by deconvolution of RNA-seq data. Genome Med. 11 (1), 34. 10.1186/s13073-019-0638-6 31126321 PMC6534875

[B9] Hu J. Yu A. Othmane B. Qiu D. Li H. Li C. (2021). Siglec15 shapes a non-inflamed tumor microenvironment and predicts the molecular subtype in bladder cancer. Theranostics 11 (7), 3089–3108. 10.7150/thno.53649 33537076 PMC7847675

[B10] Kamoun A. de Reyniès A. Allory Y. Sjödahl G. Robertson A. G. Seiler R. (2020). A Consensus molecular classification of muscle-invasive bladder cancer. Eur. Urol. 77 (4), 420–433. 10.1016/j.eururo.2019.09.006 31563503 PMC7690647

[B11] Li B. Severson E. Pignon J. C. Zhao H. Li T. Novak J. (2016). Comprehensive analyses of tumor immunity: Implications for cancer immunotherapy. Genome Biol. 17 (1), 174. 10.1186/s13059-016-1028-7 27549193 PMC4993001

[B12] Li T. Fu J. Zeng Z. Cohen D. Li J. Chen Q. (2020). TIMER2.0 for analysis of tumor-infiltrating immune cells. Nucleic Acids Res. 48 (W1), W509–w14. 10.1093/nar/gkaa407 32442275 PMC7319575

[B13] Liu B. Xiao J. Dong M. Qiu Z. Jin J. (2020). Human alkaline ceramidase 2 promotes the growth, invasion, and migration of hepatocellular carcinoma cells via sphingomyelin phosphodiesterase acid-like 3B. Cancer Sci. 111 (7), 2259–2274. 10.1111/cas.14453 32391585 PMC7385342

[B14] Morad G. Helmink B. A. Sharma P. Wargo J. A. (2021). Hallmarks of response, resistance, and toxicity to immune checkpoint blockade. Cell 184 (21), 5309–5337. 10.1016/j.cell.2021.09.020 34624224 PMC8767569

[B15] Necchi A. Raggi D. Gallina A. Ross J. S. Farè E. Giannatempo P. (2020). Impact of molecular subtyping and immune infiltration on pathological response and outcome following neoadjuvant pembrolizumab in muscle-invasive bladder cancer. Eur. Urol. 77 (6), 701–710. 10.1016/j.eururo.2020.02.028 32165065

[B16] Newman A. M. Liu C. L. Green M. R. Gentles A. J. Feng W. Xu Y. (2015). Robust enumeration of cell subsets from tissue expression profiles. Nat. Methods 12 (5), 453–457. 10.1038/nmeth.3337 25822800 PMC4739640

[B17] Parveen F. Bender D. Law S. H. Mishra V. K. Chen C. C. Ke L. Y. (2019). Role of ceramidases in sphingolipid metabolism and human diseases. Cells 8 (12), 1573. 10.3390/cells8121573 31817238 PMC6952831

[B18] Perry D. K. Obeid L. M. Hannun Y. A. (1996). Ceramide and the regulation of apoptosis and the stress response. Trends Cardiovasc Med. 6 (5), 158–162. 10.1016/1050-1738(96)00044-8 21232290

[B19] Powles T. Durán I. van der Heijden M. S. Loriot Y. Vogelzang N. J. De Giorgi U. (2018). Atezolizumab versus chemotherapy in patients with platinum-treated locally advanced or metastatic urothelial carcinoma (IMvigor211): A multicentre, open-label, phase 3 randomised controlled trial. Lancet 391 (10122), 748–757. 10.1016/S0140-6736(17)33297-X 29268948

[B20] Rosenberg J. E. Hoffman-Censits J. Powles T. van der Heijden M. S. Balar A. V. Necchi A. (2016). Atezolizumab in patients with locally advanced and metastatic urothelial carcinoma who have progressed following treatment with platinum-based chemotherapy: A single-arm, multicentre, phase 2 trial. Lancet 387 (10031), 1909–1920. 10.1016/S0140-6736(16)00561-4 26952546 PMC5480242

[B21] Ru B. Wong C. N. Tong Y. Zhong J. Y. Zhong S. S. W. Wu W. C. (2019). Tisidb: An integrated repository portal for tumor-immune system interactions. Bioinformatics 35 (20), 4200–4202. 10.1093/bioinformatics/btz210 30903160

[B22] Sassoli C. Pierucci F. Zecchi-Orlandini S. Meacci E. (2019). Sphingosine 1-phosphate (S1P)/S1P receptor signaling and mechanotransduction: Implications for intrinsic tissue repair/regeneration. Int. J. Mol. Sci. 20 (22), 5545. 10.3390/ijms20225545 31703256 PMC6888058

[B23] Sharma P. Retz M. Siefker-Radtke A. Baron A. Necchi A. Bedke J. (2017). Nivolumab in metastatic urothelial carcinoma after platinum therapy (CheckMate 275): A multicentre, single-arm, phase 2 trial. Lancet Oncol. 18 (3), 312–322. 10.1016/S1470-2045(17)30065-7 28131785

[B24] Siegel R. L. Miller K. D. Fuchs H. E. Jemal A. (2022). Cancer statistics, 2022. CA Cancer J. Clin. 72 (1), 7–33. 10.3322/caac.21708 35020204

[B25] Sjödahl G. Abrahamsson J. Bernardo C. Eriksson P. Höglund M. Liedberg F. (2022). Molecular subtypes as a basis for stratified use of neoadjuvant chemotherapy for muscle-invasive bladder cancer-A narrative Review. Cancers (Basel). 14 (7), 1692. 10.3390/cancers14071692 35406463 PMC8996989

[B26] Spiegel S. Cuvillier O. Edsall L. Kohama T. Menzeleev R. Olivera A. (1998). Roles of sphingosine-1-phosphate in cell growth, differentiation, and death. Biochem. (Mosc) 63 (1), 69–73. 9526097

[B27] Sun W. Jin J. Xu R. Hu W. Szulc Z. M. Bielawski J. (2010). Substrate specificity, membrane topology, and activity regulation of human alkaline ceramidase 2 (ACER2). J. Biol. Chem. 285 (12), 8995–9007. 10.1074/jbc.M109.069203 20089856 PMC2838321

[B28] Van Brocklyn J. R. Williams J. B. (2012). The control of the balance between ceramide and sphingosine-1-phosphate by sphingosine kinase: Oxidative stress and the seesaw of cell survival and death. Comp. Biochem. Physiol. B Biochem. Mol. Biol. 163 (1), 26–36. 10.1016/j.cbpb.2012.05.006 22613819

[B29] Wang Y. Zhang C. Jin Y. Wang He Q. Liu Z. (2017). Alkaline ceramidase 2 is a novel direct target of p53 and induces autophagy and apoptosis through ROS generation. Sci. Rep. 7, 44573. 10.1038/srep44573 28294157 PMC5353723

[B30] Witjes J. A. Bruins H. M. Cathomas R. Compérat E. M. Cowan N. C. Gakis G. (2021). European association of urology guidelines on muscle-invasive and metastatic bladder cancer: Summary of the 2020 guidelines. Eur. Urol. 79 (1), 82–104. 10.1016/j.eururo.2020.03.055 32360052

[B31] Xu L. Deng C. Pang B. Zhang X. Liu W. Liao G. (2018). Tip: A web server for resolving tumor immunophenotype profiling. Cancer Res. 78 (23), 6575–6580. 10.1158/0008-5472.CAN-18-0689 30154154

[B32] Xu R. Garcia-Barros M. Wen S. Li F. Lin C. L. Hannun Y. A. (2018). Tumor suppressor p53 links ceramide metabolism to DNA damage response through alkaline ceramidase 2. Cell Death Differ. 25 (5), 841–856. 10.1038/s41418-017-0018-y 29229990 PMC5943524

[B33] Xu R. Jin J. Hu W. Sun W. Bielawski J. Szulc Z. (2006). Golgi alkaline ceramidase regulates cell proliferation and survival by controlling levels of sphingosine and S1P. Faseb J. 20 (11), 1813–1825. 10.1096/fj.05-5689com 16940153

[B34] Xu R. Wang K. Mileva I. Hannun Y. A. Obeid L. M. Mao C. (2016). Alkaline ceramidase 2 and its bioactive product sphingosine are novel regulators of the DNA damage response. Oncotarget 7 (14), 18440–18457. 10.18632/oncotarget.7825 26943039 PMC4951300

[B35] Zhang S. Huang P. Dai H. Li Q. Hu L. Peng J. (2020). TIMELESS regulates sphingolipid metabolism and tumor cell growth through Sp1/ACER2/S1P axis in ER-positive breast cancer. Cell Death Dis. 11 (10), 892. 10.1038/s41419-020-03106-4 33093451 PMC7581802

[B36] Zheng J. Jiang X. Jiang K. Yan Y. Pan J. Liu F. (2022). miR-196a-5p correlates with chronic atrophic gastritis progression to gastric cancer and induces malignant biological behaviors of gastric cancer cells by targeting ACER2. Mol. Biotechnol. [Epub ahead of print]. 10.1007/s12033-022-00589-8 36513872

